# Light emitting diodes for the improvement of postharvest quality of wild rocket in soilless and soil-bound cultivation systems

**DOI:** 10.1016/j.heliyon.2024.e39052

**Published:** 2024-10-10

**Authors:** Martina Loi, Silvana De Leonardis, Giuseppina Mulè, Francesco Serio, Benedetta Bottiglione, Costantino Paciolla, Alessandra Villani

**Affiliations:** a Institute of Sciences of Food Production, National Research Council of Italy, Via G. Amendola, 122/O, 70126, Bari, Italy; b Department of Bioscience, Biotechnology and Environment, University of Bari Aldo Moro, Via E. Orabona 4, 70125, Bari, Italy

**Keywords:** Wild rocket, Light emitting diodes, Postharvest storage, Antioxidant components, Microbiological quality, Blue light

## Abstract

Wild rocket (*Diplotaxis tenuifolia* (L.) DC cv. Dallas) is a leafy green vegetable appreciated for its pungent taste and healthy properties, often consumed as a ready-to-eat product. The cultivation system is crucial in determining the overall quality, while postharvest storage is fundamental for preserving nutritional quality, phytochemicals, and vitamins. This study aimed to investigate the phytochemical content and microbiological quality of soilless (SS) and soil-bound (SB) wild rocket during cold postharvest storage under blue, red, and green Light Emitting Diode (LED). Blue LED increased chlorophylls and carotenoids in SB after two days of storage, and chlorophyll *a* in SS after seven days. Furthermore, it reduced H_2_O_2_ levels after two days (SS and SB) and lipid peroxidation in SB. Red LED increased phenols in both SS and SB but was detrimental to chlorophyll, carotenoids, and oxidative markers. Green LED had less significant effects. Microbiological growth varied with LED treatment: green light increased mesophilic bacteria in SB, and red light did so in SS by day four, while blue light reduced bacterial growth at the end of storage. Overall, Blue LED was the most effective LED in preserving postharvest quality. Soilless cultivation was particularly beneficial in reducing lipid peroxidation and maintaining cell membrane integrity during long-term storage, and it might also be more effective in preserving ascorbic acid. Conversely, soil-bound cultivation methods could enhance initial polyphenol content or better preserve it during early storage. This study highlights the complex interplay of pre-harvest conditions, postharvest quality, and shelf-life performance.

## Abbreviations

ASC: Ascorbic Acid
BL: Blue Light Emitting Diode
DM: Dry Matter
FW: Fresh Weight
GL: Green Light Emitting Diode
H_2_O_2_: Hydrogen Peroxide
LED: Light Emitting Diode
MDA: Malondialdehyde
RL: Red Light Emitting Diode
SB: Soil-Bound
SL: Soilless

## 1 Introduction

Wild rocket (*Diplotaxis tenuifolia* (L.) DC cv. Dallas) is a leafy green vegetable belonging to the Brassicaceae family, widely known for its pleasant pungent taste and health-promoting phytonutrients, like minerals, vitamins, and antioxidant compounds [1]. Besides its organoleptic and nutritional properties, wild rocket is appreciated for its fast growth in different climatic conditions, adaptability, ease of propagation, and its –two to five growing cycles per season [2].

Wild rocket is often consumed raw in minimally processed, ready-to-eat salads together with other leafy vegetables. Its processing generally includes only sorting, washing, rinsing, and packaging in modified atmosphere. Therefore, it is important that the visual, nutritional, and microbial qualities are maintained throughout the shelf-life.

Pedoclimatic factors including light, temperature, and soil/water salinity, along with cultural practices like cultivation system, nutrient, and water management, and genotype, i.e. species and cultivar, are among the most important factors determining the postharvest quality and shelf-life of fresh products [3]. Together with the traditional soil-bound (SB) culturing technique, soilless cultivation (SS) is being increasingly used for the cultivation of wild rocket and other leafy vegetables, with the aim of fulfilling the high year-round demand and obtain higher yield and higher quality. Indeed, SS cultivation allows to manage nutrient composition and electrical conductivity, the dissolved oxygen concentration, while the control of temperature and humidity specifically lowers the risk of microbiological spoilage [4,5].

After harvest, several physiological processes take place, such as respiration, ripening, and senescence. These processes can significantly impact the quality and shelf-life of fresh products, like wild rocket, because they lead to water loss, softening, ripening, and loss of nutritional value [6]. Additionally, the storage time of such products largely depends on the presence of microorganisms that can proliferate during storage and contribute to quality loss [7].

Light intensity, distribution, photoperiod, and spectra have been shown to greatly impact the physiology and quality of leafy and non-leafy vegetables in the field, after harvest and during storage [8]. Among the different light systems that can be applied, light emitting diodes (LED) show unique advantages in terms of selective spectra emission, higher efficiency, higher lifespan, lower cost, and low heat dissipation with respect to traditional fluorescent lamps, alongside the possibility to dim [9,10]. Selective spectra emission allows the selective activation of specific light-sensing pathways, which can trigger desired physiological response in the target plant, while minimizing unwanted effects.

LED application enables to influence the shelf-life and quality of fresh products by modulating weight loss, senescence, ripening, and enhancing the production of antioxidant compounds [9].

In a previous study [11] the effect of nutrient supply level and growth system on physiological responses, quality, and antioxidant systems of wild rocket at harvest was assessed.

Recently, Villani and colleagues [11] evaluated for the first time the effect of different nutrient supply levels and cultivation systems on growth, antioxidant components, quality, and mineral content in *D. tenuifolia* cv. Dallas, highlighting that this cultivar responded strongly to the cultivation method. Nonetheless, the impact of the cultivation system and LEDs application in the postharvest storage of wild rocket grown under different conditions still needs to be investigated. Red, blue, and white LEDs can maintain the quality of most vegetables during the postharvest period, promote phytochemical accumulation, and delay senescence. Blue LED light enhances the synthesis of beneficial compounds like flavonoids and anthocyanins and improves photosynthesis. However, since the effects vary by plant species, cultivar, and organs, specific assessments are necessary [8,9].

Therefore, the aim of this work was to investigate the effect of three different LED (blue, green, and red) and darkness on the post-harvest quality of wild rocket (*Diplotaxis tenuifolia* (L.) DC) cultivated in soil and soilless conditions.

## 2 Materials and methods

During the storage at 4 °C, antioxidants content, including ascorbic acid, polyphenols, chlorophylls and carotenoids, parameters of oxidative stress, such as lipid peroxidation and hydrogen peroxide, and microbiological load were evaluated with the aim to determine the best LED spectra for wild rocket post-harvest storage and to study the impact of the growth system on LED response.

### 2.1 Plant material and storage conditions

In a recent study [11], wild rocket cv. Dallas (Isi Sementi, Fidenza, PR. Italy) was cultivated in SB and SS growing systems using two input fertilization programs: low and high.

The same leaves harvested in that study were utilized in our current investigation, which focuses on the effects of different postharvest light treatments on phytochemical content and microbiological quality. The experiment was carried out at the “La Noria” experimental farm of the Institute of Sciences of Food Production – National Research Council (Mola di Bari, Italy, 41°03′ N; 17°04′ E; 24 m above sea level). Wild rocket (cv. Dallas, Isi Sementi, Fidenza, PR. Italy) plants were cultivated in two independent sectors equipped for soil-bound and for soilless cultivation. Leaves of plants growing in SB and SS under low fertilization program (N dose of 30 kg ha^−1^ for SB and a nutrient solution containing 11.0 mM of N for SS) were harvested between April 28th and May 10th, 2021, when reaching commercial length of 10–12 cm. After each harvest time the fresh-cut rocket leaves were immediately transported in refrigerate conditions to the Postharvest laboratory. Freshly cut wild rocket leaves were carefully selected, discarding those physically damaged, dehydrated, or yellowed, and 7.0 ± 0.1 g of leaves were placed in 250 mL in polypropylene trays (10 × 8 × 3.5 cm), distributed over one only layer, to allow a uniform radiation and avoid dehydration. The trays were packaged in pierced freezer plastic bags and immediately stored in the domestic refrigerator (Panasonic mod. NRBN34FW1) at 4 °C and 85 % RH.

### 2.2 Postharvest LED light treatments

LED lights (OSRAM GmbH, Germany), consisting of linear modules of five LEDs each connected in parallel, were disposed on the top of each refrigerator's shelf, illuminating 24 h throughout the shelf-life period with a photon flux density (PPFD) of 20 ± 2.5 μmol m^−2^ s^−1^ (Supplementary Fig. 1). The PPFD was measured using a Delta OHM Photo-Radiometer (mod. HD2302.0, Pordenone, Italy). Four treatments were applied, as reported in Ref. [12]:

- Darkness (D) storage was used as Control
- Blue (BL): monochromatic blue LED with a peak at 467 nm
- Green (GL): monochromatic green LED with a peak at 522 nm
- Red (RL): monochromatic red LED with a peak at 625 nm

After two, four, and seven days of cold storage, samples were collected for quality analyses. Three repetitions per light treatment and day of analysis were prepared. On sampling days, samples were removed from the trays, weighed, and immediately frozen in liquid nitrogen. Then, they were freeze-dried and stored at −80 °C until further analysis, except for samples used for dry matter and microbial analyses.

### 2.3 Determination of dry matter content

For dry matter (DM) content measurement, 15 g of fresh wild rocket leaves were dried at 60 °C in an oven for 48 h or until a constant weight was obtained. DM was then calculated as the percentage of the ratio of the dry weight to the fresh matter.

### 2.4 Chlorophylls and carotenoids content

One gram of leaves was homogenized with 15 mL of 95 % ethanol and centrifuged at 20,000×*g* for 15 min. The absorbance of supernatant was measured at 645 and 662 for chlorophyll *a* and *b*, respectively, and at 470 nm for carotenoids. Chlorophyll (*a*, *b*, and *a*:*b* ratio) and carotenoids contents were calculated according to Ref. [13] and expressed as mg kg^−1^ fresh weight.

### 2.5 Determination of ascorbic acid and total phenolic content

The contents of ascorbic acid (ASC) and total phenolic were assayed according to Ref. [14] and expressed as mg ASC kg^−1^ fresh weight and mg of gallic acid equivalents g^−1^ fresh weight, respectively.

### 2.6 H_2_O_2_ content and lipid peroxidation level

Frozen leaf samples (1 g) were ground in liquid nitrogen and added with 0.1 % thiobarbituric acid (TCA). The details of extraction processes and the measurements of absorbances at 390 nm and 532 nm for evaluating H_2_O_2_ and MDA content, respectively, were described in Refs. [12,15].

### 2.7 Microbial quality

Ten grams-samples were transferred to sterile stomacher bags, added with 90 mL sterile saline solution and homogenized for 1.5 min in a stomacher (Seward, London, UK). After 20 min of incubation at room temperature, the suspension was decimally diluted. Aliquots of 100 μL were spread-plated or onto count plates (Merck, Darmstad, Germany) of selective media following the manufacturers’ instructions. Total Viable Count (mesophiles) was determined on Plate Count Agar (PCA, Difco, 30 °C, 48 h), *Pseudomonas* spp. On Pseudomonas Agar Base (PAB, Oxoid Ltd; 25 °C, 48 h), yeasts and fungi on Potato Dextrose Agar (PDA, Oxoid Ltd; 25 °C, 48–96h).

### 2.8 Statistical analysis

A two-way repeated measures ANOVA was used to assess the significance of light treatments, cultivation method, and their interaction effect. Data were examined for normality of distribution using the D'Agostino-Pearson test to determine whether they required transformation before performing ANOVA. If ANOVA showed significant differences, Tukey's test was used to determine differences between treatments. Statistical analyses were performed with using GraphPad Prism software version 9.0.0 for Windows, GraphPad Software, San Diego, California USA. Reported data are mean values out of at least three replicates of three independent experiments. The error bars in all figures represent the standard deviation of the means.

## 3 Results

### 3.1 Dry weight

The initial DM content of the samples was 12.12 ± 0.5 % for SB and 8.57 ± 0.31 % for SS. At harvest and during the cold storage SB samples showed higher DM content than the SS cultivated rocket (Fig. 1). No significant dry matter change was shown for all light treatments in SB compared to the dark, except for RL at seven days, where a significant decrease was observed. The same trend was reported for SS leaves, although at seven days of storage both RL and GL significantly (*P* < 0.05) decreased the DM content. The DM was found to be significantly affected by the cultivation method, light qualities and their interactions (Fig. 1).

**Fig. 1:**
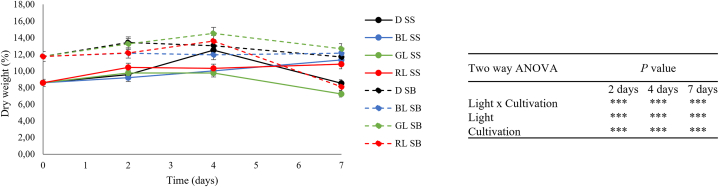
Relative weight loss (% from initial weight) of wild rocket leaves grown in soilless (SS) and soil (SB) during 2, 4, and 7 days of storage at 4 °C in response to LEDs in different spectral regions (blue, green, and red) and darkness as a control. ∗ Significant differences at *P* < 0.05 by comparing values under light conditions against samples in darkness. Data represent the mean (±SD) of at least 3 replicates.

### 3.2 Chlorophylls and carotenoid content

Total chlorophyll content at the harvest time was higher in SB wild rocket leaves than in SS (Table 1).

**Table 1:** Chlorophyll (*a*, *b*, and *a*:*b* ratio) and carotenoids levels, as influenced by exposure to LED light after two and seven days of cold storage.

**Sample**	**days**	**Chl *a***	**Chl *b***	**Total Chl**	**Chl *a*:*b***	**Carotenoids**
		**(mg kg**^**−**^**^1^)**	**(mg kg**^**−**^**^1^)**	**(mg kg**^**−**^**^1^)**		**(mg kg**^−1^**)**
SS	T0	260.2 ± 3.8	140.1 ± 1.3	400.3 ± 0.56	1.86	200.2 ± 2.29
SB	T0	581.7 ± 14.2	91.3 ± 3.9	672.9 ± 17.1	6.37	186.5 ± 4.5
DL SS	2	442.3 ± 1.62 d	278.6 ± 3.5 g	720.9 ± 5.12 e	1.59	282.8 ± 2.40 d
BL SS	2	316,4 ± 3.1 a	158.9 ± 0.1 e	475.4 ± 3.1 a	1.99	193.9 ± 5.5 b
GL SS	2	377,3 ± 3.8 c	189.3 ± 8.8 f	566.7 ± 12.6 c	1.99	217.7 ± 5.3 c
RL SS	2	341,0 ± 0.8 b	158.5 ± 0.3 e	499.5 ± 0.5 b	2.15	191.1 ± 1.5 b
DL SB	2	631.7 ± 7.7 g	105.3 ± 6.7 c	736.9 ± 6.7 e	6.00	204.9 ± 2.1 bc
BL SB	2	765.5 ± 9.2 h	126.1 ± 1.8 d	891.5 ± 7.8 f	6.07	231.2 ± 16.6 c
GL SB	2	579.8 ± 0.2 f	83.62 ± 0.1 b	663.4 ± 0.1 d	6.93	194.8 ± 3.1 b
RL SB	2	483.2 ± 0.8 e	70.91 ± 0.3 a	554.1 ± 0.5 c	6.81	161.4 ± 0.8 a
						
DL SS	7	627.8 ± 5.87 c	139.1 ± 22.1 c	766.9 ± 16.2 cd	4.51	176.1 ± 2.66 b
BL SS	7	679.5 ± 34.3 d	120.5 ± 11.7 bc	800.2 ± 42.6 d	5.64	223.7 ± 12.8 b
GL SS	7	622.3 ± 12.8 c	104.6 ± 4.0 b	726.9 ± 16.8 bc	5.95	202.9 ± 3.9 b
RL SS	7	564.6 ± 9.0 b	101.1 ± 0.5b	665.8 ± 9.5 b	5.58	187.5 ± 2.7 a
DL SB	7	660.6 ± 7.6 cd	107.3 ± 1.3 b	767.9 ± 8.9 cd	6.15	204.8 ± 2.5 c
BL SB	7	642.0 ± 5.6 cd	81.5 ± 0.7 ab	723.5 ± 6.2 bc	7.87	214.4 ± 2.1 c
GL SB	7	563.3 ± 14.6 b	118.1 ± 13.3 bc	681.4 ± 1.3 c	4.77	181.3 ± 3.8 b
RL SB	7	458.7 ± 24.6 a	63.2 ± 7.4 a	521.9 ± 31.9 a	7.25	145.4 ± 6.5 a
						
Two way ANOVA		***P* value**				
Source of variation 2 days		Chl *a*	Chl *b*	Total Chl		Carotenoids
Light x Cultivation		∗∗∗	∗∗∗	∗∗∗		∗∗∗
Light		∗∗∗	∗∗∗	∗∗∗		∗∗∗
Cultivation		∗∗∗	∗∗∗	∗∗∗		∗∗∗
						
Source of variation 7 days						
Light x Cultivation		∗∗∗	∗∗∗	∗∗∗		∗∗∗
Light		∗∗∗	∗∗∗	∗∗∗		∗∗∗
Cultivation		∗∗∗	∗∗∗	∗∗∗		∗∗∗

Mean values ± SD are shown for n = 5. Statistical analyses were performed for each time point. The letters indicate groups that are significantly different from each other (*p* < 0.05). Groups sharing the same letter are not significantly different. Wild rocket leaves grown in soilless (SS) and soil-bound (SB) conditions.

In SS, after two days of storage, the chl levels decreased by 34 %, 21 %, and 31 % on average under BL, GL, and RL treatment, respectively, as compared to darkness. Specifically, the chl *a* and *b* contents decreased during two days of storage, with BL and RL resulting in the lowest levels of chl *b* (43 % less than control). At the end of the storage (seven days), RL decreased the total chlorophyll in all treatments both for chl *a* and chl *b* contents (*ca.* 20 % less than the control). A significant (*P* < 0.05) accumulation of chlorophylls was induced by the exposure to BL in SB leaves at two days (*ca.* 32 %), while in SS leaves only chl *a* increased (8 %) after seven days (*P* < 0.05). After two days, in SS leaves stored under BL and RL total chl values were significantly lower compared to other samples, while SB leaves exposed to BL showed the highest value. After seven days, SS leaves stored under BL showed the highest total chlorophyll content and the value was significantly different from all samples except those stored into the dark. In SS samples, chl *a*:*b* ratio increased under all light treatments at both time of storage compared to the control. Similarly, in SB leaves, RL increased chl *a*:*b* ratio after two days by an average of 14 % and after seven days by an average of 18 %, compared to the dark. However, higher chl *a*:*b* ratio was maintained under green light treatment only after two days, whereas only after seven days SB leaves treated with blue light resulted in increased (28 %) chl *a*:*b* ratio (Table 1). After two days, carotenoids content decreased in SS leaves by an average of 31 % (BL), 23 % (GL), and 32 % (RL). However, in SB leaves RL decreased carotenoid concentrations by an average of 21 %, but the content was unaffected (GL) or had a 21 % increase under BL. After seven days, SB leaves exposed to RL showed the lowest carotenoid content, while those exposed to BL and DL showed the highest carotenoid content. Similarly, SS leaves stored under RL showed a significant lower value compared to the highest treatments. Overall, these results suggest that both the type of light treatment and the cultivation method, as well as their interaction, significantly influence the levels of chlorophylls and carotenoids in rocket leaves.

### 3.3 Ascorbic acid and polyphenols contents

**Fig. 2:**
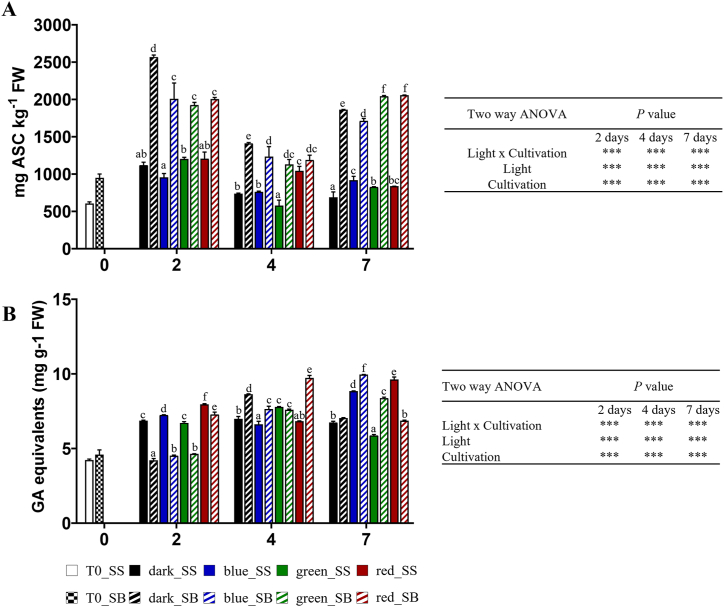
A) Ascorbic acid (ASC) and B) polyphenols contents in wild rocket leaves grown in soilless (SS) and soil (SB) during 2, 4, and 7 days of storage at 4 °C in response to LEDs in different spectral regions (blue, green, and red) and darkness. Any two means within a column not followed by the same letter are significantly different at *P* < 0.05 by ANOVA. Data represent the mean (±SD) of at least 3 replicates. FW: fresh weight.

On day two of refrigerated storage, the rocket leaves cultivated in soil exhibited significantly higher ascorbic acid (ASC) content compared to those grown in soilless (Fig. 2A). By day four, a similar trend was observed, with the soil-bound grown leaves maintaining higher ASC levels than their soilless counterparts stored under BL, GL, and into the dark. Same trend was observed after seven days, suggesting that the method of cultivation plays a crucial role in preserving ascorbic acid content during early storage. Nonetheless, the soil-bound grown leaves still demonstrated significantly higher ASC levels compared to the soilless leaves.

Polyphenols content increased under RL at two and seven days in the SS leaves and at two and four days in the SB leaves (Fig. 2B). On day two of refrigerated storage, the rocket leaves cultivated into the soil showed significantly higher polyphenol content, compared to those grown in soilless, as indicated by different letters (*p* < 0.05). On day four, the trend persisted with soil-bound grown leaves maintaining significantly higher GA levels than the soilless leaves, indicating a sustained effect of the cultivation method on polyphenol preservation. By day seven, the SB leaves stored under BL and GL continued to exhibit significantly higher polyphenol levels compared to the SS leaves, highlighting the potential advantage of SB cultivation in preserving polyphenol content during prolonged storage under these light treatments. Overall, both conditions (light and cultivation method) and their interaction significantly affect the content of ascorbic acid and polyphenols in the rocket leaves.

### 3.4 Effect of light treatment on H_2_O_2_ content and lipid peroxidation level

**Fig. 3:**
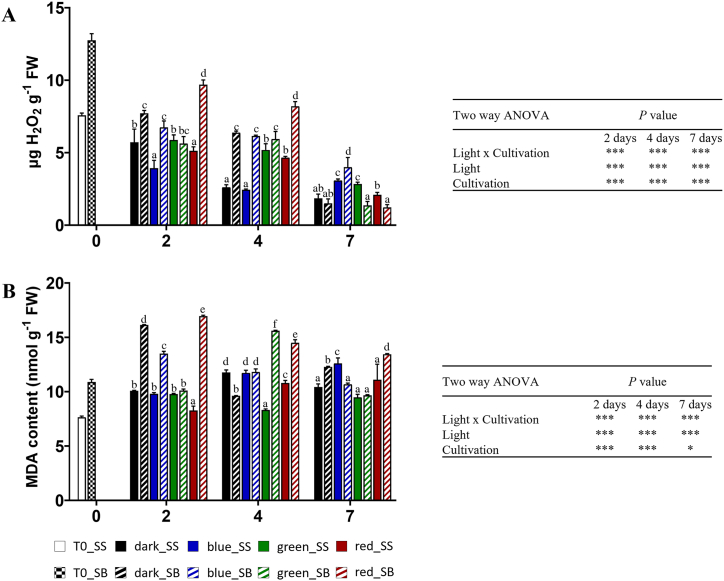
A) Hydrogen peroxide (H_2_O_2_) and B) malondialdehyde (MDA) contents in wild rocket leaves grown in soilless (SS) and soil (SB), during 2, 4, and 7 days of storage at 4 °C in response to LEDs in different spectral regions (blue, green, and red) and darkness. Any two means within a column not followed by the same letter are significantly different at *P* < 0.05 by ANOVA. Data represents the mean (±SD) of at least 3 replicates. FW: fresh weight.

The level of some markers for oxidative stress, such as hydrogen peroxide (H_2_O_2_) and MDA, was evaluated and results are shown in Fig. 3. As showed in Fig. 3A, on day two of refrigerated storage, rocket leaves cultivated in soilless systems and stored in the dark (dark_SS) exhibited significantly lower hydrogen peroxide (H_2_O_2_) content compared to those grown in soil and stored in the dark (dark_SB). Similarly, leaves stored under BL, RL, and DL also showed significant differences, with the soilless cultivation resulting in lower H_2_O_2_ levels than soil cultivation in both cultivation conditions. By day four, the trend continued with SS leaves maintaining significantly lower H_2_O_2_ levels than SB leaves. Moreover, both at two and four days RL caused the highest level of H_2_O_2_ in leaves grown in soil. By day seven, although H_2_O_2_ levels decreased in all groups, SB leaves stored under BL showed the highest level of H_2_O_2_.

On day two of refrigerated storage, the rocket leaves cultivated without soil and stored in the dark showed significantly lower malondialdehyde (MDA) content, a marker of lipid peroxidation, compared to those grown in soil and stored in the dark (Fig. 3B). Similarly, the SS leaves stored under BL and RL exhibited significantly lower MDA levels than SB leaves. By day four, SB leaves exposed to GL and RL showed higher MDA levels compared to the other treatments. After seven days, SS leaves still had significantly lower MDA content compared to SB leaves under dark and red treatments, while BL and RL enhanced the MDA level in SS and SB leaves, respectively. Overall soilless cultivation and green light minimized lipid peroxidation and accumulation of H_2_O_2_ content and the interaction of both light treatment and cultivation method influenced the oxidative stress in rocket leaves during refrigerated storage (Fig. 3A and B).

### 3.5 Effect of light treatment on microbial quality

**Fig. 4:**
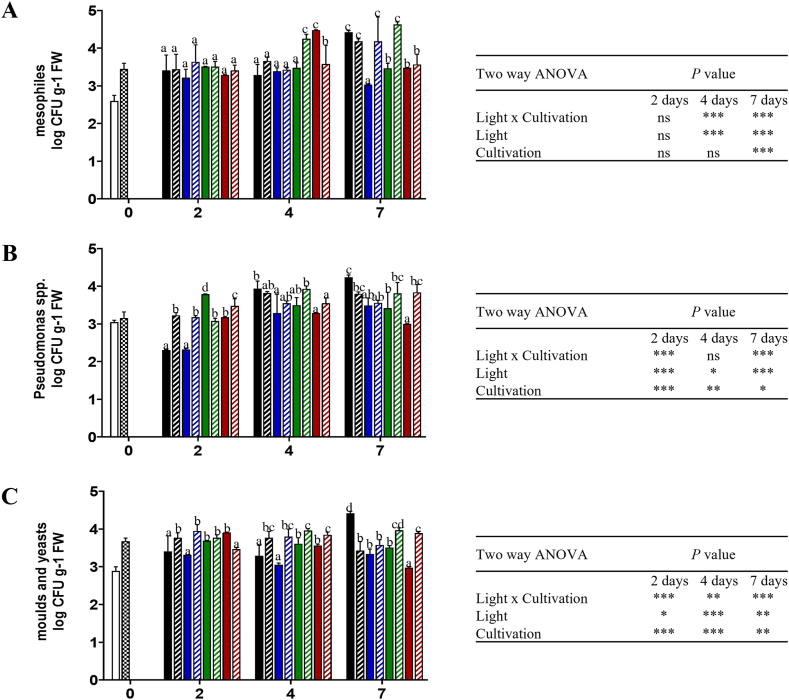
Microbial loads of mesophyles (A), moulds and yeasts (B), and Pseudomonas spp (C) in wild rocket leaves grown in soilless (SS) and soil (SB) during 2, 4, and 7 days of storage at 4 °C in response to LEDs in different spectral regions (blue, green, and red) and darkness. Any two means within a column not followed by the same letter are significantly different at *P* < 0.05 by ANOVA. Data represents the mean (±SD) of at least 3 replicates. FW: fresh weight.

The microbiological growth was affected by LED light treatments, as showed in Fig. 4.

As reported in Ref. [11], the SB leaves showed higher levels of microbial counts compared to the SS leaves. However, cultivation method did not affect mesophiles count during storage (except after seven days), while it influenced fungi and *Pseudomonas* spp. accumulation at each time of storage (Fig. 4A–C). Mesophilic bacteria showed a significant higher growth under GL (SB leaves) and RL (SS leaves) at day four, while BL significantly decreased mesophilic bacteria growth at the end of storage (Fig. 4A). *Pseudomonas* spp. growth significantly decreased under BL and DL regimes at two days, whereas moulds and yeasts growth decreased under RL treatment at two days and increased in GL and RL treated samples after seven days (Fig. 4B). Similarly, BL significantly decreased moulds and yeasts content compared to the other light treatments, maintaining lower levels also at the end of storage (Fig. 4C). Overall, light and cultivation significantly influenced the microbiological growth, except for the mesophilic bacteria growth at day two and four, while the interaction of both factors was not significant only for the mesophilic bacteria growth after two days and for the *Pseudomonas* spp. growth after four days (Fig. 4A–C).

## 4 Discussion

*D. tenuifolia* cv. Dallas is one of the most popular leafy vegetables grown in the Mediterranean area due to its health and nutritional properties. Different growing systems are currently used for the cultivation of wild rocket, namely SB and SS. The cultivation system, together with other preharvest factors, is important in determining the growth and development of plants, the nutritional quality, the minerals, phytochemicals, and vitamins content [3,11]. Moreover, cultivation management greatly affects the quality of fresh products also during the shelf-life [3,16,17]. As also reported in a previous study [11], wild rocket leaves grown in SS cultivation showed lower chlorophylls, ASC, H_2_O_2_ and MDA content, higher phenols, and microbiological quality than SB. During cold postharvest storage under LEDs, SS and SB samples did not show univocal behaviors, highlighting that the growing system influences the plant response to different postharvest storage conditions, such as light. Bonasia et al. [2] studied the post-harvest performance of wild rocket genotypes grown in different periods and with different soilless cultivation systems. They found that the improved nutritional quality was attributed to increased temperature and sunlight, cultivation system, and moderate electrical conductivity.

Several studies showed that LED spectra and intensity can influence the shelf-life and quality of fresh produce, reducing weight loss, senescence, and over-ripening, and enhancing the production of antioxidant compounds (ASC, polyphenols, and pigments) [9,18]. Photoreceptors are proteins able to perceive and transduce light signals in the cell activating specific signaling pathways. Specific light wavelengths can be perceived by chromophore molecules which are integral parts of photoreceptors, namely cryptochromes (blue), phytochromes (red), and opsin (green). The physiological responses induced by light include vegetative growth, differentiation, circadian rhythm, and secondary metabolism [9].

Postharvest light application, especially BL, is reported to increase weight loss due to stomatal openings and increased transpiration [19,20]. Nonetheless, in our samples significant weight loss did not occur until four days, and, overall, wild rocket leaves stored under light treatments were comparable to those stored in dark conditions. This trend can be explained by an endogen cell control aimed to maintain a basal metabolism of the leaves. The strong decrease in dry matter occurred at seven days in presence of RL in SB, and RL and GL in SS, can be due to a larger use of storage compounds for longer time and/or higher active metabolism in leaves. Additionally, the simultaneous decrease of chl levels suggests that lower photosynthates are produced.

In this study, leaves grown in SB showed an increase in ASC content during the storage time, likely to counteract the increased level of H_2_O_2_. Low intensity BL was reported to up-regulate chlorophyll-related genes expression during the postharvest storage in several vegetables, including wild rocket [19], and to extend the duration of active photosynthesis and photosynthetic capacity [8]. Our result confirmed this evidence, showing an increase of chls in SB. In addition, the concurrent increase in carotenoids under BL, that are part of light-harvesting complex, underlines their fundamental protective role from photo-oxidative damages of the chls.

The increase of phenols with RL treatment in both SS and SB cultivation systems indicates that specific monochromatic RL wavelength of 625 nm could be useful to improve quality and enhance these antioxidant compounds, improving the shelf-life of wild rocket leaves. This suggests that RL has a higher impact on phenols than the cultivation system. The observed MDA and H_2_O_2_ values, although higher than control in some instance, are still comparable with those obtained in previous studies [11,21].

A clear positive effect of LED irradiation on the microbiological quality could be observed only in SS with all lights, especially after seven days. BL and RL have been extensively reported to reduce the microbial load and the production of toxic secondary metabolites [22]. Different wavelengths within the visible spectrum have been reported to effectively reduce microbial growth on vegetables during refrigerated storage [19,23], including UV and Far-RL, which were not assayed in this study [24]. So far, a consistent conclusion on the effect of LEDs on microbial growth has not been disclosed, though it has been reported that LED can induce cell damage, targeting photosensitive endogenous compounds such as porphyrins and flavins [9]. The study of the interaction between the plant bioactive compounds and the microbial community during post-harvest storage deserves further investigations because it plays a pivotal role in determining the quantity and quality of microorganisms.

The results of this study confirm that the relationship between pre- and post-harvest interventions and the nutritional quality, phytochemical contents, senescence, and oxidative stress responses of rocket leaves is governed by complex biochemical and molecular mechanisms which still need to be fully unravelled [19,25]. The use of LED in the postharvest storage may foster beneficial effects in terms of antioxidants content and microbiological quality of wild rocket leaves. Nonetheless, LED exerts different metabolic perturbations even on the same cultivar grown using two different cultivations systems, like SS and SB. BL had and increasing effect on chlorophylls and carotenoids in SB, while showed the opposite effect in SS leaves, except after seven days, where BL showed the highest total chlorophyll content. Other positive effects exerted by BL were on H_2_O_2_ after two and four days on SS leaves. RL increased phenols both in SS leaves (day two and seven) and SB leaves (day two and four), H_2_O_2_ and MDA in SB leaves, nevertheless it was deleterious for chlorophyll and carotenoids content. On the other hand, GL elicited less significant effects on the other tested parameters with respect to the dark control. Based on these results, BL was generally the most effective LED to preserve the post-harvest quality of wild rocket and could be used to improve quality in terms of antioxidant compounds (chlorophyll, carotenoids, ascorbic acid, polyphenols). Overall, the observed differences in the response to LEDs can be attributed to the different antioxidant status of wild rocket leaves at harvest, depending also on the growth system, able to differently support the metabolic activities during the postharvest storage.

## 5 Conclusion

The results of this study contribute to describing the complex interplay of pre-harvest conditions, postharvest quality, and shelf-life performance. Indeed, agronomic, biochemical, and transcriptomic studies underline how phytochemical contents, and their expression also varies among pre- and post-harvest treatments, or even among different breeding lines under controlled environmental conditions. Hence, more data are needed to fill this knowledge gap and further explore the potential of crop improvement, enhanced shelf-life, and nutritional quality. These results underscore the advantage of soilless cultivation in minimizing lipid peroxidation and maintaining cell membrane integrity during extended storage, regardless of the light conditions. Soilless cultivation may be also more effective in retaining ascorbic acid over a longer storage period. Soil-bound cultivation methods might enhance the initial polyphenol content or better preserve it during early storage.

## Funding

This research was funded by Sus&Low-Sustaining Low-Impact Practices In Horticulture Through Non-Destructive Approach To Provide More Information On Fresh Produce History And Quality; grant number: 201785Z5H9” – CUP B74I19001120005 and by the European Union – Next Generation EU - National Recovery and Resilience Plan (PNRR) - Mission 4 Component 2 Investment 1.4 - Call for proposals No. 3138 of 16/12/2021 of Italian Ministry of University and Research; Project code CN00000022, Project title " National Research Centre for Agricultural Technologies” (Agritech)”, Concession Decree No. 1032 of 17/06/2022 adopted by the Italian Ministry of University and Research, CUP D93C22000420001. This work was supported by the University of Bari Aldo Moro, grant number H96J15001610005 and by Research for Innovation (REFIN)—POR PUGLIA FESR-FSE 2014/2020—codice progetto 7B942221—CUP H94I20000410008.

## CRediT authorship contribution statement

**Martina Loi:** Writing – review & editing, Writing – original draft, Visualization, Investigation. **Silvana De Leonardis:** Visualization, Investigation. **Giuseppina Mulè:** Writing – review & editing, Writing – original draft, Supervision. **Francesco Serio:** Writing – review & editing, Validation, Formal analysis. **Benedetta Bottiglione:** Writing – review & editing, Formal analysis. **Costantino Paciolla:** Writing – review & editing, Supervision, Funding acquisition, Conceptualization. **Alessandra Villani:** Writing – review & editing, Writing – original draft, Visualization, Project administration, Investigation, Funding acquisition, Formal analysis, Conceptualization.

## Data and code availability

Data included in article/supplementary material is referenced in the article.

## Data availability statement

The authors declare that they have no known competing financial interests or personal relationships that could have appeared to influence the work reported in this paper.

## Declaration of competing interest

The authors declare that they have no known competing financial interests or personal relationships that could have appeared to influence the work reported in this paper.

## Figures and Tables

**figs1:**
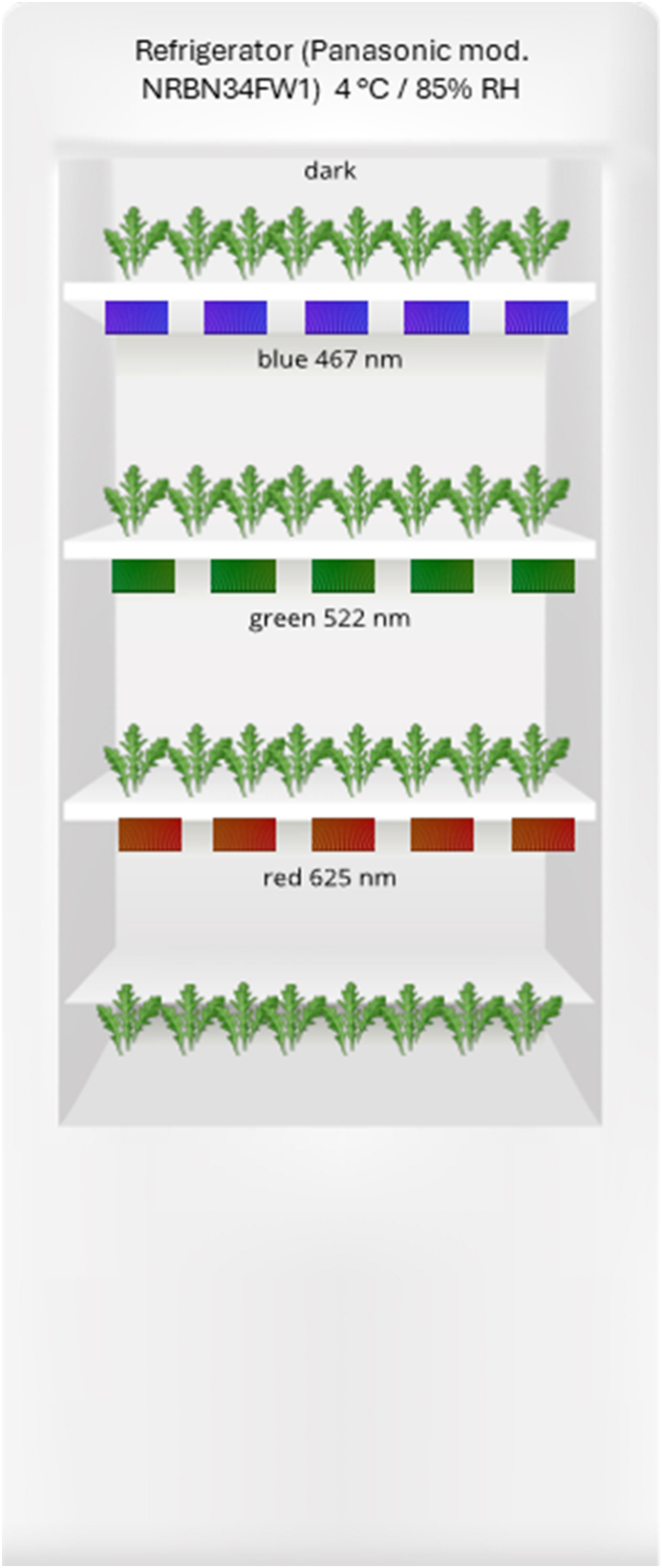

